# Impact of Different Oak Chips’ Aging on the Volatile Compounds and Sensory Characteristics of *Vitis amurensis* Wines

**DOI:** 10.3390/foods11081126

**Published:** 2022-04-14

**Authors:** Yanxia Yu, Lingxi Li, Ruowei Xue, Chen Wang, Mengying Chen, João Ramos, Shuting Zhang, Baoshan Sun

**Affiliations:** 1 School of Traditional Chinese Materia Medica, Shenyang Pharmaceutical University, Shenyang 110016, China; yuyanxia2022@163.com (Y.Y.); xuerw1995@163.com (R.X.); 2 School of Functional Food and Wine, Shenyang Pharmaceutical University, Shenyang 110016, China; lingxilee@163.com (L.L.); chenwang2067@163.com (C.W.); cmyhengzhi@126.com (M.C.); zstzwz-3@163.com (S.Z.); 3 Departamento de Enologia, Herdade do Esporão, Reguengos de Monsaraz, 7200-999 Évora, Portugal; joaotramos@gmail.com; 4 Pólo Dois Portos, Instituto National de Investigação Agrária e Veterinária, I.P., Quinta da Almoinha, 2565-191 Dois Portos, Portugal

**Keywords:** *Vitis amurensis* wine, oak chips, aging, sensory characteristics

## Abstract

In this work, different oak chips were used to age *Vitis amurensis* wine, and the effects on sensory properties were observed. Twenty-one different oak chips were added to a one-year-old wine made by a traditional technique. The wine was aged for 6 months before analysis by CIELab for color parameters, GC–MS for volatile compounds, and electronic tongue and a tasting panel for sensory properties. The results showed that the addition of any tested oak chip could significantly strengthen the wine’s red color. Among 61 volatile compounds, alcohols presented the highest concentrations (873 to 1401 mg/L), followed by esters (568 to 1039 mg/L) and organic acids (157 to 435 mg/L), while aldehydes and volatile phenols occurred at low concentrations. Different oak species with different toasting levels could affect, to varying degrees, the concentrations of esters, alcohols, and volatile phenols, but to a lesser extent those of aldehydes. Sensory analysis by a tasting panel indicated that non- and moderately roasted oak chips gave the wines higher scores than those with heavy toasting levels. The major mouthfeel descriptors determined by electronic tongue were in good agreement with those from the tasting panel.

## 1. Introduction

*Vitis amurensis* Rupr. is an East Asian member of the Vitaceae family. It originates from China and is distributed mainly in China, Russia, and Korea [1]. Because it is one of the most cold-tolerant grape varieties, it has been studied extensively [2,3,4]. Berries of *V. amurensis* have been used in the wine industry in Northeastern China for more than 70 years. Studies have found that the active constituents, i.e., the polyphenols, and the antioxidant properties of *V. amurensis* wine are 2 to 16 times and 5 to 15 times higher, respectively, than those of *V. vinifera* wine [5,6]. In addition, *V. amurensis* grape berries contain a wide range of nutrients, suggesting that this species could provide excellent raw materials for wine-making [3]. However, berry skins from *V. amurensis* grapevines have a high tannin content, resulting in wines with a strongly astringent mouthfeel [7]. It is, therefore, important to establish a method for improving the quality of *V. amurensis* wine. The wine quality is predominantly affected by its sensory properties (color, aroma, and taste). In order to make quality dry red wine, producers use various methods, for example, delaying the picking time, girdling at different periods, root restriction, malolactic fermentation, low-temperature treatment, and aging processes [8,9,10].

Aging in oak barrels is a traditional winemaking practice, providing the wine with volatile oak aroma compounds and oak polyphenols, thus improving its quality [11,12,13]. After barrel aging, the wine usually shows fewer vegetal notes and higher complexity with a new aroma profile [14,15]. At the same time, wood pores can gently oxidize some compounds, resulting in a reduction in astringency and changes in color [14]. Since wine aging in barrels is slow and expensive, the use of oak chips has been proposed as a valid alternative for accelerating and reducing the cost of producing wood-flavored wine. Wine aging in the presence of oak chips has exhibited a higher production of aroma compounds and hydrolyzed tannins, increasing the quality of the wine [14,16]. Puech et al. found that oak contains 40~45% cellulose, 20~25% hemicellulose, 25~30% lignin, and 8~15% tannin [17,18,19].

Oak chips of different origins with different toasting levels have different effects on the sensory characteristics of the wine. If the features of the wine do not integrate well with the oak elements, the wine will lose its specific characteristics. There is no clear stipulation on what kind of oak treatment is suitable for a particular type of wine, so the oak treatment must be carefully selected.

The objective of this work was to improve the quality of *V. amurensis* wine by aging it with oak chips. For this purpose, different kinds of oak chips, namely, non-toasted French oak (NFr), moderately roasted French oak (MFr), heavily roasted French oak (HFr), moderately roasted Chinese oak (MCh), heavily roasted Chinese oak (HCh), moderately roasted American oak (MAm), as well as the combination of any two of these, were tested. The CIELab method was used for the analysis of the color parameters, GC–MS analysis for quantification of the volatile compounds, and electronic tongue and a tasting panel analysis for the evaluation of the sensory properties of the tested wines.

## 2. Materials and Methods

### 2.1. Materials

Chips of heavily toasted French oak, moderately toasted French oak, non-toasted French oak, and moderately toasted American oak were purchased from Enartis (Beijing, China). The chips of moderately toasted Chinese oak (Quercus mongolica) and heavily toasted Chinese oak (Quercus mongolica) were provided by Fisch. ex Ledeb (Jilin, China).

Ethyloctanoate, 1-pentanol, propane-1, 1,3-triethoxy, 3-ethoxypropanol, 1-octene-3-ol, phenylethyl alcohol, pentadecanoic acid, 3-methyl butyl ester, and n-decanoic acid were obtained from Chengdu Chroma-Biotechnology Co., Ltd. (Chengdu, China). The pure hydrocarbon mixture (C10-C23) standard was obtained from Chengdu Chroma-Biotechnology Co., Ltd. All chemicals and reagents were obtained from Tianjin Chemical Company, Ltd. (Tianjin, China).

### 2.2. Preparation of Vitis Amurensis Wine

*Vitis amurensis* grapes (Shuang Hong variety) cultivated on the Zijinggege estate (Jian, Jilin, China) were harvested in the technological ripeness stage during the vintage period (September–October) of 2016. The *Vitis amurensis* wine was made by the winery of the same estate on an industrial scale using traditional vinification technology. The harvested grape clusters were crushed and destemmed using a destemmer-crusher. The must was collected in stainless steel tanks and treated with sulfur dioxide (50 mg/L) before undergoing alcoholic fermentation at 25°C. The cap was punched down twice a day until it remained submerged. After six days of maceration, when alcoholic fermentation was finished, the wine was pressed. Free-run and press wines were combined and stored in a stainless steel tank at 25°C. The racking treatments were performed at the end of three, six, and twelve months of wine storage. After each racking, sulfur dioxide (30 mg/L) was added. The wine stored for one year was then divided into various 2 L micro-stainless-steel tanks for further aging with oak chips. The *Vitis amurensis* wine before the oak-chip aging experiments presented the following physico-chemical characteristics: alcohol content 10.68 (% vol), total sugar 3.63 g/L, dry extract 31.60 g/L, total acidity 16.17 g/L (expressed as tartaric acid), volatile acidity 0.50 g/L (expressed as acetic acid), free SO_2_ 30 mg/L, and total SO_2_ 130 mg/L.

### 2.3. Oak-Chip Aging

The experimental oak-chip aging conditions are reported in Table 1. The tested chips include those of heavily, moderately, and non-toasted French oak, moderately toasted American oak, heavily toasted Chinese oak, moderately toasted Chinese oak, as well as the combination of any two of these. Prior to further analysis, a total of 21 different oak chips were added individually in different micro-stainless steel tanks and aged at 15°C for six months.

### 2.4. Determination of Polyphenols

#### 2.4.1. Determination of Total Phenolic Compounds

The total phenolic content (TP) was measured using the modified Folin–Ciocalteau method [20,21]; 0.2 mL of samples were diluted 5 times and mixed with 8 mL of 7.5% sodium carbonate. After 5 min, 0.5 mL of 2 N Folin–Ciocalteau reagent was added, and the volume was adjusted to 10 mL using water. Next, the color (absorbance) generated after about 120 min at 25°C was measured at 760 nm. Gallic acid was used to construct a calibration and expressed as gallic acid equivalent (GAE).

#### 2.4.2. Determination of Total Tannins

In this study, the total tannin content (TTA) of the *V. amurensis* wines was examined following the previously reported phenanthroline spectrophotometry method with appropriate modifications [22]. The TTA was measured spectrophotometrically using tannic acid as reference. Then, the standard solution with different concentration gradients was diluted 5 times with 10% ethanol. Ammonium ferric sulfate was added to the standard solution and allowed to react at 80°C for 25 min. Then, buffer solution, 1,10-phenanthroline monohydrate, and EDTA were added one after the other. Lastly, the absorbance was measured at 442 nm.

### 2.5. Color Evaluation

The WSC-3B CIELab (Shanghai Inesa Optical Instrument Co., LTD., Shanghai, China) tristimulus colorimeter was used to record the wine color values, such as L* (lightness), a* (red/green values), b* (yellow/blue values), c* (chroma), and h* (hue angle). ΔE* (color difference) was used for a comprehensive measurement of color. The L* axis represented the wine lightness scale, which ranged from 0 to 100; L* = 0 means black, while L* = 100 means white. The a* value represents the degree of red and green, and the higher the value of a*, the more the color tends toward red. Similarly, the higher the value of b*, the more it tends toward yellow. The c* value represents the color saturation. The larger the value of c*, the higher the color saturation. The value of the hue angle (h*) ranged from 0° to 360°, with red wine generally being between 0° and 90°. Lower values of h* lead to purple or ruby red, while higher values lead to brick red or reddish-brown. ΔE* represents the difference in the comprehensive color of the sample.

### 2.6. Extraction and GC–MS Analysis of Aroma Components

The aroma components of each wine sample were extracted by liquid–liquid extraction in accordance with Yin et al. [23]. Briefly, 5 mL of wine samples were extracted three times with dichloromethane at a ratio of 1:1. The extracts were combined and concentrated to 5 mL, then filtered and analyzed by GC–MS (Thermo Trace 1300-ISQ; Thermo Technology Co., Ltd., Maltham, MA, USA). The oven temperature was programmed at an initial temperature of 40°C for 10 min, increased at a rate of 3°C/min to 160°C, further increased up to 240°C at a rate of 6°C/min, and maintained at this temperature for 25 min. The carrier gas was helium (99.996%) at a flow rate of 1 mL/min followed by a 1:75 split ratio. The temperature of the injection port was 260°C. Mass spectrometry detection was performed by electronic impact ionization (70 eV). The temperatures used were 260°C for the trap and 255°C for the transfer line, and the scan range was from 50 to 650 amu.

The internal standard was prepared by dissolving the accurate transfer reference standard of 2-octanol in dichloromethane to yield concentrations of 8.3 mg/mL. Standard solutions were prepared by dissolving the accurate transfer reference standard of 1-pentanol, propane-1,1,3-triethoxy, 3-ethoxypropanol, ethyloctanoate, 1-octene-3-ol, phenylethyl alcohol, pentadecanoic acid,3-methyl butyl ester, and n-decanoic acid in dichloromethane to yield concentrations of 816, 900, 904, 878, 837, 1020, 865, and 886 μg/mL of the stock solution. An appropriate amount of stock solution was taken at the concentrations of 244.8, 135.0, 135.6, 52.7, 251.1, 306.0, 259.5, and 265.7 μg/mL and diluted step by step to concentrations of 7.650, 4.219, 4.238, 1.646, 7.847, 9.562, 8.109, and 8.304 μg/mL to obtain the mixed standard solution. Quantitative standards and calibration curves for the quantification of volatile compounds are presented in Supplementary Tables S1 and S2.

The identification of the volatile compounds was confirmed by comparing their mass spectra (HP MSD chemical workstation and NIST08 spectrum library) and their retention times with those of the pure compounds. The compounds of existing standards were quantified by the internal standard method, and the compounds without standards were quantified by reference materials with similar chemical structures and functional groups.

### 2.7. Sensory Analysis by Electronic Tongue

Electronic tongue (e-tongue) (SA402B multi-channel bionic lipid membrane electronic tongue, Intelligent Sensor Technology, Inc., Kanagawa, Japan) was used for taste measurement, according to previous reports [24]. The detection system consists of six electrochemical sensors (AAE, CTO, Cao, AE1, COO, and GL1) and a reference electrode (Ag/AgCl). The main taste attributes of each sensor are: AAE sensor (umami), CTO sensor (saltiness), Cao sensor (sourness), AE1 sensor (astringency), COO sensor (bitterness), and GL1 sensor (sweetness). In addition to the above five taste senses, the electronic tongue system can also detect the aftertaste of bitterness and astringency through the potential difference. The electrodes were connected to a multi-frequency and large-amplitude pulse scanner controlled by a computer. The e-tongue analysis was conducted immediately after opening the wine bottle, and 15 mL of each sample was poured into the measuring cup for testing. The working electrode was cleaned between each measurement to prevent any cumulative effects. The results were subjected to principal component analysis and radar graph analysis.

#### 2.7.1. Principal Component Analysis (PCA)

Principal component analysis (PCA), as a commonly used method of data dimensionality reduction, can transform multiple indexes representing multiple characteristics of samples into 2–3 comprehensive indexes. There is no relationship between these comprehensive indicators, but it can reflect the information of the original multiple indicators. These indicators are then transformed into a new coordinate system, and the PCA diagram is obtained. The smaller the distance between the samples on the PCA diagram, the closer the sample; the larger the distance on the PCA diagram, the greater the characteristic difference. The distance can characterize the difference between the samples.

#### 2.7.2. Radar Graph Analysis

*Vitis amurensis* wines with different oak chips have different tastes. The radar graph can clearly reflect the taste values of all kinds of *V. amurensis* wines, which is convenient for comparison and analysis. In this study, the effect of oak-chip aging on the richness, astringent aftertaste (After-A), bitter aftertaste (After-B), sourness, sweetness, bitterness, astringency, umami, and saltiness of the wines were analyzed.

### 2.8. Sensory Evaluation by Tasting Panel

Sensory evaluation of the 6-month-aged wines with oak chips was performed by a tasting panel composed of 12 trained judges who had Wine & Spirit Education Trust (WSET) Level 3 Award in Wines qualifications and participated regularly in wine-tasting sessions. Standard glasses of wine for tasting (NFV09-110) were used. Other tasting conditions were as follows: room temperature, 20°C; wine temperature, 16°C–18°C; amount of wine, a quarter to a third of the volume of the glass. The process of the sensory evaluation included observing the appearance under suitable light. To judge the aroma, the taster sniffs the wine at rest for 5–8 s, then shakes the glass to smell the aroma for 5–10 s, with an interval of 1–2 min between the two sniffs. The taster then sips 6–10 mL of wine. The amount should be the same each time so that the wine covers the tongue. While inhaling a small mouthful of air, the taster closes the lips, stirs the tongue, feels for 12–15 s, spits out the wine sample, feels the wine taste for 5–8 s, and the sample tasting is over. The taster then gargles with distilled water and continues to the next wine after the feeling disappears completely. The wine is scored using the Wine Tasting Table (AWS) of the Wine Institute of America as the evaluation index, and several specific descriptors for the aromatic profile of wine are referred to on the Wine Aroma Wheel (U.C. Davis Aroma Wheel). It is scored from five aspects: appearance, aroma, taste and structure, aftertaste, and overall impression, while the total score is calculated after averaging each evaluation index. The 20-point method was used in Table 2 [25].

### 2.9. Statistical Analysis

Vinification and oak-chip aging experiments were performed in replicate and sample analysis in triplicate. The average values and standard deviations were calculated using Excel 2010 software. The SPSS 17.0 software was used for statistical analysis, and analysis of variance was used to assess significance. The heat map was made using the R studio 3.6.3 software. The PCA plot was made using the matlab 7.0 software.

## 3. Results

### 3.1. Polyphenol Content of V. amurensis Wines

The total polyphenol and tannin contents in *V. amurensis* wines before and after aging are shown in Table 3. Based on the analysis of the content of polyphenol compounds in the wine samples, the tannin contents of the wine increased significantly after oak aging. The total polyphenol content of the wine ranged from 7.89 to 9.43 g/L, and the total tannin content to be tested was between 4.57 g/L and 6.18 g/L. It can be seen from Table 3 that the total polyphenol and tannin contents in the wine increased after aging, which may be due to increased hydrolyzed tannins [26,27]. There was no significant difference in the total polyphenol and tannin contents of samples treated with French oak with different roasting levels, and the same was true for Chinese oak. The MCh:HCh sample had the highest polyphenol content. In addition, the total polyphenol content in wines aged with Chinese oaks was higher than that of wines treated with American and French oaks.

### 3.2. Color Evaluation

Table 4 shows that there were significant differences in the color parameters among the *V. amurensis* wines before and after oak-chip aging. It was observed that the wines darkened (lower L*) after aging, which would be due to their higher phenolic content. The a* value and h* value had significant differences, while the b* and ΔE* value had no significant differences before and after aging. The addition of oak chips increased the red hue of the wine. The more colorful the red wine, the better its appearance. Except for the *V. amurensis* wine with oak chips MFr:MCh, which changed to a yellow hue, the b* of the other aged wines did not change significantly. The results show that the color saturation of oak-chip-aged wines was improved. In addition, the h* value of the red wine was between 0° and 90°, and the color changed to ruby red. There were significant differences in the color intensity between the aged *V. amurensis* wine and the control group.

### 3.3. Aroma Components

**Figure 1:**
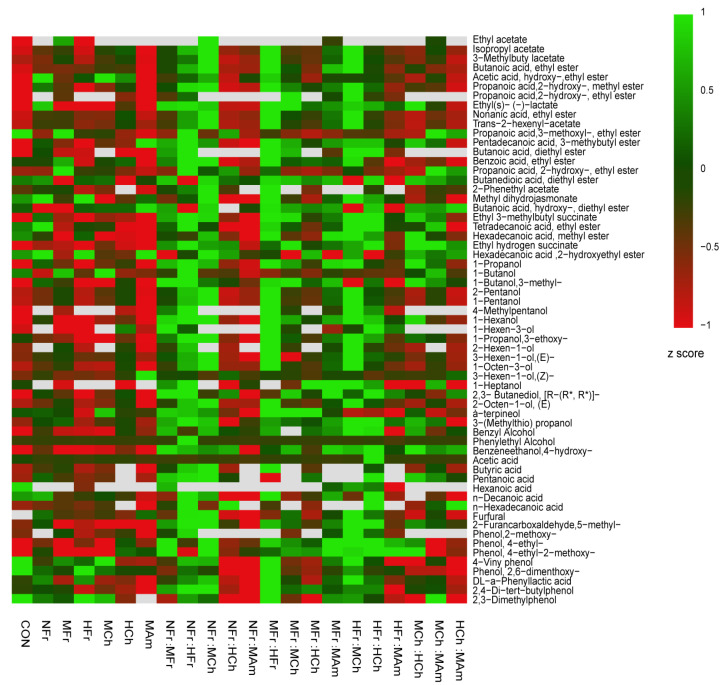
Heatmap representation of the GC–MS-determined aroma content of compounds in wines aged with different oak chips. In normalized mapping, the negative value is lower than the average value of all numbers, and the aroma content increases from red to green.

The compounds for which the standards were available were quantified by the internal standard method, and the compounds without standards were quantified using compounds with similar chemical structures and functional groups as references. The contents of the quantified aromatic compounds are presented in Table 5. According to Table 5, a total of 24 esters, 21 alcohols, 6 acids, 2 aldehydes, and 8 volatile phenols were detected in nearly all tested wines aged with different oak chips. However, the quantified aroma-component contents were varied among the different oak-chip-aged wines. Figure 1 presents a heat map representing the aroma composition data of different oak chips and combinations. Through the heat map, the content of the aroma components can be expressed by color, and the change in contents can be clearly seen. We can observe that after aging, the main components of aroma components, i.e., esters and alcohols, have increased. It seems that the effect of single aging was not as good significant as that of mixed aging, and the increasing quality trend of NFr:HFr, NFr:MCh, NFr:MFr, MFr:MAm, HFr:MCh is more obvious.

#### 3.3.1. Esters

Esters give wines their primary fruit and floral aromas and contribute substantially to the flavor of wine [28]. Ester molecules are compounds formed by the condensation of a hydroxyl group of a phenol or alcohol and a carboxyl group from an organic acid. As one of the most important volatile constituents in grape wine, esters also directly influence the aromatic profiles and sensory perception of wines. In this study, a total of 24 esters were detected, most of which were acetate esters and ethyl esters of fatty acids. It was notable that the ester contents increased significantly after aging. In 21 oak-chip-treated samples, the ester compounds presenting high content were isopropyl acetate, ethyl lactate, pentadecanoic acid, 3-methylbutyl ester, butanoic acid, hydroxy-, diethyl ester, and ethyl hydrogen succinate. The isopropyl acetate content in the wine aged with MFr:HFr increased by more than two times, while the ethyl lactate content increased between 3.42% and 42.55% after aging. Butanoic acid and diethyl ester increased by between 11.85% and 71.30%. The change in ester contents due to aging may provide rich flower and fruit fragrances for the wines.

#### 3.3.2. Alcohols

Alcohols are generally considered to be the aromatic compounds with the greatest impact on the aroma of wine [29,30]. Excessive concentrations of alcohols can result in a strong, pungent smell and taste, whereas optimal levels impart fruity characteristics [31]. A total of 21 alcohol compounds were detected in this study, and there was an overall increase in the alcohol contents of the *V. amurensis* wine after oak-chip aging (Table 5). The increase in alcohol content was more pronounced for the wines aged with NFr:MFr, NFr:HFr, NFr:MCh, MFr:HFr, and MFr:MAm. It was found that 3-methyl-1-butanol and phenylethyl alcohol were abundant in the wine, and studies have shown that they have cheese, honey, and rose aromas, respectively [32]. In addition, 1-pentanol and 1-hexanol were also detected. While 1-pentanol is known to have a mellow flavor, 1-hexanol is said to taste of grass and toast [33]. When these compounds are combined, the flavors in the wine may change, making the aroma of *V. amurensis* wine more complex and layered.

#### 3.3.3. Acids

The organic acids in wine come primarily from the berries (grapes) and are precursors for the synthesis of esters, which can increase the mellowness of wine. Moreover, organic acids have preservative properties and increase the physical and chemical stability of wine. It has been reported that, at appropriate levels, the organic acids play an important role in the aromatic equilibrium of wine, mainly because they restrict the hydrolysis of the relevant esters and maintain a high content of aromatic esters [34]. Having an appropriate amount of organic acid is important in *V. amurensis* wine. The acetic acid content is high and usually constitutes about 90% of the volatile acids in wine [35]. Six organic acids were identified in this study. After aging with oak chips, the organic acid content in *V. amurensis* wine decreased significantly, thus alleviating the high acidity of the product. A previous study considered that decanoic acid (fatty and unpleasant notes) negatively affected the overall wine aroma [34]. We found small amounts of decanoic acid in *V. amurensis* wine. In addition, the hexanoic acid content, which smells of cat urine and sweat, decreased significantly with oak-chip aging. A low concentration of hexanoic acid was only detected in the wines aged with HFr, NFr:MFr, MFr:MAm, HFr:MCh, HFr:HCh, and HFr:MAm.

The overall content of the eight volatile phenols detected in this study was not high and increasing or decreasing trends were not apparent. We found that 4-viny phenol showed no significant change after aging, consistent with previous research results [36]. Another important volatile compound detected in oak-chip-aged *V. amurensis* wine was furfural, which might result from the decomposition of pentose, mainly from hemi-cellulose in oak chips. The increased furfural might add fragrance, fruit, and flower aromas to the *V. amurensis* wine [32].

In summary, after oak-chip aging, the total aroma component contents in 21 kinds of aged *V. amurensis* wine increased. Specifically, the content of alcohols and esters increased significantly, while the content of organic acid compounds decreased, which may have been due to the esterification reaction. The highest concentrations of volatile compounds were found in the wines aged with MFr:HFr, NFr:HFr, and NFr:MCh up to 3.011 g/L, 2.863 g/L, and 2.905 g/L. However, some of the aroma components occurred at low levels, and, combined with other minor compounds, may provide delicate background aromas that contribute to the complexity and equilibrium of the overall varietal aroma. At the same time, it can be seen from Figure 1 that, NFr:HFr, NFr:MCh, MFr:MAm, and HFr:MCh have an overall aging effect, and the effect of mixed aging is more pronounced than that of single aging.

### 3.4. Sensory Evaluation by Panelists

Table 6 shows the sensory evaluation results for the *V. amurensis* wines aged with different kinds of oak chips. After the treatment with oak chips, the total scores of the sensory evaluation were higher than those of the control, which meant a total sensory quality promotion. As well as the control, the wine treated with HFr, HCh, and MAm oak chips received a lower sensory evaluation score than others due to the poor taste and structure as well as an inadequate aftertaste. The wines treated with NFr:HFr, NFr:MCh, and MFr:MAm oak chips obtained the highest scores, with a clear, shiny body, typical varietal aromas, fresh fruity flavors, and a good, balanced aftertaste. According to the test panel, the wines aged in contact with MAm added “vanilla” and “toast” aromas. Moreover, the wines aged with NFr, MFr, and MCh also had rich aromas, with some “vanilla”, “toast”, and “smoky” aromas added, but they were not as obvious as those of MAm-treated wine. It was found that HFr and HCh were too heavy to cover their fruit aromas. At the same time, there were some pleasant, toasted-nut aromas in the heavily toasted group. In addition, the addition of mixed oak chips enriched the aroma but also produced some adverse effects. Notably, MAm:NFr had a better aftertaste, and MAm:HCh had a longer aftertaste. Among the four groups of MAm, the MCh:MAm and HCh:MAm produced some less-pleasant smells of overripe fruit, with MAm:HCh being slightly more astringent. Among the five groups treated with HFr, MAm, MCh, and MFr had a better performance. The wine samples treated with HFr:MFr had rich chocolate and fruit flavors.

### 3.5. Electronic Tongue (E-Tongue) Evaluation

#### 3.5.1. Principal Component Analysis (PCA)

**Figure 2:**
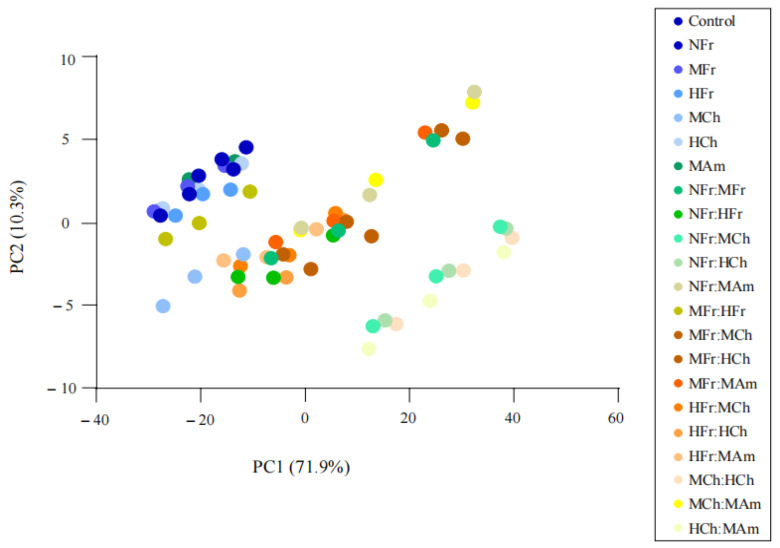
PCA plot of the electronic tongue detection results of wines of *Vitis amurensis*.

The principal component analysis clearly distinguished 21 kinds of aged *V. amurensis* wines, indicating that the e-tongue could evaluate the taste differences of the oak-chip aged wines to some extent (Figure 2). The first two principal components possessed 82.2% of the total variance (71.9% and 10.3% for PC1 and PC2), which showed that these factors were sufficiently important to warrant further discussion. As can be seen from Figure 2, 22 kinds of *V. amurensis* wines aged with different oak chips can be divided into three groups. Among them, the control and *V. amurensis* wines with NFr, MFr, HFr, MCh, and MAm oak chips as the first group; *V. amurensis* wines with HCh, NFr:MFr, NFr:HFr, NFr:MAm, MFr:HFr, MFr:MCh, MFr:HCh, MFr:MAm, HFr:MCh, HFr:HCh, HFr:MAm, and MCh:MAm oak chips as the second group; and the rest as the third group. The distinction between these three groups of *V. amurensis* wines was obvious. Among the six kinds of *V. amurensis* wines aged with just one type of oak chip, except for the *V. amurensis* grapes with heavily roasted Chinese oak chips (HCh), the rest of the *V. amurensis* wines were in the same group as the control wine. Although these wines were quite similar to the control, they were also slightly different. The HCh and mixed-oak-chip aging tended to converge, which may be because high-temperature toasting changes the polyphenol composition and affects the flavor. The group comprising NFr:MCh, NFr:HCh, MCh:HCh, and HCh:MAm were probably Chinese oak with a strong flavor. In addition to these groups, other oak chip combinations with Chinese oak may mask some of the oak and toasty flavors of the Chinese oak.

#### 3.5.2. Radar Graph of *V. amurensis* Wines

**Figure 3:**
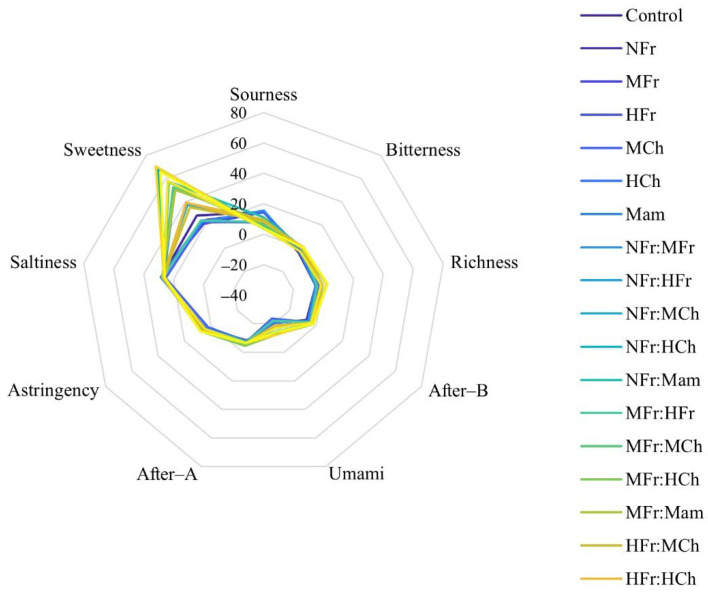
Taste radar graph of wine made from *Vitis amurensis*.

The e-tongue taste radar graph of *V. amurensis* wines with different oak chips is shown in Figure 3. The obtained data from the electronic tongue evaluation are presented in Supplementary Table S3. There was no significant difference in saltiness, astringent aftertaste (After-A), and bitter aftertaste (After-B) between the control and the other 21 oak-chip-aged wines. Compared with the control wine, it was found that the acidity of oak-chip-aged *V. amurensis* wines decreased. The sweet taste decreased in single-oak-chip-aged wines but increased in mixed-oak-chip-aged ones. The same was true for the umami taste. The e-tongue results generally supported the wine panel’s results. In the sensory evaluation by the panel, sweet and strong tastes were also detected. The mixed-oak-chip aging increased the complexity of the wines, covered up some bad smells, and also covered up some of the fruit aromas. However, the wines aged with single oak chips had a fruity aroma, which was not as layered as that of the wines aged with mixed oak chips.

## 4. Discussion

As shown in Table 3, the total polyphenol and tannin contents in all tested wines increased after oak-chip aging. Liu et al. studied the effect of oak chips on wine quality and found that the content of polyphenols increased after oak aging [37], which may be due to the increase in hydrolyzed tannins from the oak chips [26,27]. In the evaluation of color parameters (Table 4), the values of a* showed a downward trend, which was consistent with a study by Perez-Magarino et al., who found that red tones fell (values of a*) with aging. They explained that the loss of red tones is mainly due to the loss of free anthocyanins [38]. Regarding the values of b*, except for the fact that MFr:MCh tends toward yellow, there were no significant differences. Mateus et al. pointed out that a small amount of oxygen in the wine body can oxidize ethanol to form acetaldehyde, and acetaldehyde can be involved in the formation of polymeric pigments. Then, because polymeric pigments are mostly yellow, red wines often become lighter in color during aging [39]. This result is consistent with that of Li et al. [40].

In the GC–MS analysis (Table 5), the types and concentrations of esters and alcohols in *V. amurensis* wine accounted for a large proportion of the constituents. They are among the most important volatile components, and their contribution to the flavor cannot be ignored. We noticed that ethyl acetate was not detected after some oak aging, which was inconsistent with the research of Georgiana et al. [41]. Considering the low content of other detected components, we speculated that it had reacted with other aromatic constituents. As for 4-viny phenol, which is formed by the enzymatic hydrolysis or thermal decarboxylation of cinnamic acid, it exerts a smoky aroma, which is also an indicator of the relative degree of the roasting of the oak chips because it is mainly formed by the degradation of lignin during the roasting process [41,42]. Aldehydes and acids also play important roles in supplementing and modifying the flavor of wines. An appropriate amount of acid increases the taste of wine, participates in the esterification reaction, and gives the wine a fruity aroma. Six kinds of acids were detected, and the content of acetic acid was the highest. Overall, the aroma components and contents of the wine aged with different oak chips varied, and esters and alcohols were the main aroma components. This result is generally consistent with the findings of most studies [36,41].

In the sensory evaluation by the panel, it was found that the addition of any tested oak chip could significantly strengthen the wine’s red color, i.e., from violet to ruby or garnet red, particularly when using non-toasted French oak and moderately toasted American oak. Gordillo et al. showed that the addition of oak chips promoted color enhancement and stability [43]. The oak-chip-aged wines had high sweet-taste intensity and were full-bodied. They differed from the control in that they were not as acidic and astringent, possibly due to the high sweetness intensity, which reduced the perception of acidity [44]. High levels of sweetness were also detected in the e-tongue results. Tannins gradually become softer during the aging process, and the astringency is gradually reduced. According to the test panel evaluation, the vanilla flavor of the wines might be related to a higher 1-hexanol content (Table 5), which has been reported to be responsible for the perception of a vanilla odor. In addition, 3-hexen-1-ol and trans-2-hexenyl-acetate are known to contribute to vanilla odor. Their contents were greater in the wine treated with oak chips than in the control group (Table 5). The fruit aroma in the wine is also more obvious in the aged wines than in the control. It can be seen from Table 5 that the contents of ethyl lactate and isopropyl acetate are higher in oak-chip-treated wines, providing elegant fruity and creamy flavors [36].

In summary, the mixed-oak-chip aging treatment increased the complexity of the wines, masked some bitter and astringent tastes, and also covered up some fruit aroma. However, the wine aged with single oak chips had more fruit aroma, the astringency was more obvious, and the aroma was not as layered as that in wines aged with mixed oak chips. On the basis of the above, in order to make wines of different styles, different oak chips and mixed oak chips can be selectively added, and the sensory complexity and layering can be altered by changing the oak chip treatment. If more of the fruit taste of the grape is preferred, the wine can be aged by adding single oak chips with lower toasting levels, rendering the wine softer and smoother in texture.

## 5. Conclusions

In this study, taking the color, aroma components, and taste as the main evaluation indexes, the effects of different oak-chip aging treatments on the sensory properties of *V. amurensis* wines were comprehensively analyzed. The type of oak chips should be selected according to the characteristics of *V. amurensis* wine. The aging process enhanced the organoleptic properties of the wine. A CIELab analysis showed that, after oak-chip aging, *V. amurensis* wine increased in brightness, and its color changed to ruby red. Moreover, the types of aroma components increased, with the alcohol and ester content increasing and the acid content decreasing. A combination of various aroma components gave the *V. amurensis* wine a unique flavor, taste, and aroma. The e-tongue technical analysis showed that the sour taste of *V. amurensis* wine decreased slightly with oak-chip aging, while the sweetness, astringency, freshness, and bitterness increased, and the increase in sweetness was the most obvious. After oak-chip aging, the color, aroma structure, and taste of *V. amurensis* wines were significantly improved, and mixed oak chips were observed to have the most satisfactory effects. Furthermore, the *V. amurensis* wines aged with mixed oak chips had a better appearance, aroma, and taste, with a clear and shiny body, ruby-red color, rich fruit aroma, good wood flavor, mellowness, harmoniousness, a long taste, and a rich personality. The wines aged with mixed oak chips exhibited specific characteristics and appeared to have long-aging potential.

## Figures and Tables

**Table 1:** Addition of the 21 oak chips to wines of *Vitis amurensis,* including different single oak chips and combined oak chips, dosage (4 g/L).

No.	Samples	Sample Abbreviation	Total Additive Amounts (g/L)	Sample Proportion
1	Control	Control		
2	Non-toasted French oak	NFr	4	1
3	Moderately toasted French oak	MFr	4	1
4	Heavily toasted French oak	HFr	4	1
5	Moderately toasted Chinese oak	MCh	4	1
6	Heavily toasted Chinese oak	HCh	4	1
7	Moderately toasted American oak	MAm	4	1
8	Non-toasted French oak:Moderately toasted French oak	NFr:MFr	4	1:1
9	Non-toasted French oak:Heavily toasted French oak	NFr:HFr	4	1:1
10	Non-toasted French oak:Moderately toasted Chinese oak	NFr:MCh	4	1:1
11	Non-toasted French oak:Heavily toasted Chinese oak	NFr:HCh	4	1:1
12	Non-toasted French oak:Moderately toasted American oak	NFr:MAm	4	1:1
13	Moderately toasted French oak:Heavily toasted French oak	MFr:HFr	4	1:1
14	Moderately toasted French oak:Moderately toasted Chinese oak	MFr:MCh	4	1:1
15	Moderately toasted French oak:Heavily toasted Chinese oak	MFr:HCh	4	1:1
16	Moderately toasted French oak:Moderately toasted American oak	MFr:MAm	4	1:1
17	Heavily toasted French oak:Moderately toasted Chinese oak	HFr:MCh	4	1:1
18	Heavily toasted French oak:Heavily toasted Chinese oak	HFr:HCh	4	1:1
19	Heavily toasted French oak:Moderately toasted American oak	HFr:MAm	4	1:1
20	Moderately toasted Chinese oak:Heavily toasted Chinese oak	MCh:HCh	4	1:1
21	Moderately toasted Chinese oak:Moderately toasted American oak	MCh:MAm	4	1:1
22	Heavily toasted Chinese oak:Moderately toasted American oak	HCh:MAm	4	1:1

**Table 2:** Evaluation of sensory qualities.

	Appearance, 3 Max	Aroma, 6 Max	Taste and Texture, 6 Max	Aftertaste, 3 Max	Overall Impression, 2 Max	Total Scores
Grades	3—Excellent -Brilliant with outstanding characteristic color. 2—Good -Clear with characteristic color. 1—Poor -Slight haze and or slightly off-color. 0—Objectionable -Cloudy and/or off-color.	6—Extraordinary -Unmistakable, characteristic aroma of grape variety or wine type. Outstanding and complex bouquet. Exceptional balance of aroma and bouquet. 5—Excellent -Characteristic aroma. Complex bouquet. Well balanced. 4—Good -Characteristic aroma. Distinguishable bouquet. 3—Acceptable -Slight aroma and bouquet. Pleasant. 2—Deficient -No perceptible aroma or bouquet or with slight off odors. 1—Poor -Off odors. 0—Objectionable -Objectionable or offensive odors.	6—Extraordinary -Unmistakable, characteristic flavor of grape variety or wine type. Extraordinary balance. Smooth, full-bodied, and overwhelming. 5—Excellent -All of the above, but a little less. Excellent, but not overwhelming. 4—Good -Characteristic grape variety or wine type flavor. Good balance. Smooth. May have minor imperfections. 3—Acceptable -Undistinguished wine but pleasant. May have minor off-flavors. May be slightly out of balance and/or somewhat thin or rough. 2—Deficient -Undistinguished wine with more pronounced faults than above. 1—Poor -Disagreeable flavors, poorly balanced, and/or unpleasant. 0—Objectionable -Objectionable or offensive flavors and/or texture.	3—Excellent -Lingering, outstanding aftertaste. 2—Good -Pleasant aftertaste. 1-Poor -Little or no distinguishable aftertaste. 0—Objectionable -Unpleasant aftertaste.	2—Excellent 1—Good 0—Poor	18–20 Extraordinary 15–17 Excellent 12–14 Good 9–11 Commercially Acceptable 6–8 Deficient 0–5 Poor and objectionable

**Table 3:** Total polyphenols and total tannins of wines of *Vitis amurensis.*

No.	Sample Abbreviation	TP (g/L)	TTA(g/L)
1	Control	7.91 ± 0.03d	4.57 ± 0.12c
2	NFr	8.26 ± 0.04cd	4.92 ± 0.06bc
3	MFr	8.44 ± 0.08cd	5.09 ± 0.10bc
4	HFr	8.17 ± 0.09cd	4.82 ± 0.42bc
5	MCh	8.80 ± 0.04bc	5.45 ± 0.06b
6	HCh	8.95 ± 0.03b	5.61 ± 0.05ab
7	Mam	8.54 ± 0.09c	5.20 ± 0.14bc
8	NFr:MFr	7.89 ± 0.07d	4.67 ± 0.11c
9	NFr:HFr	8.46 ± 0.18c	5.11 ± 0.37bc
10	NFr:MCh	8.66 ± 0.11bc	5.32 ± 0.17bc
11	NFr:HCh	8.91 ± 0.09bc	5.56 ± 0.14ab
12	NFr:Mam	8.36 ± 0.16cd	5.01 ± 0.23bc
13	MFr:HFr	8.43 ± 0.13cd	5.09 ± 0.19bc
14	MFr:MCh	8.35 ± 0.11cd	5.00 ± 0.17bc
15	MFr:HCh	9.36 ± 0.19a	6.02 ± 0.26ab
16	MFr:Mam	8.49 ± 0.13c	5.15 ± 0.18bc
17	HFr:MCh	7.92 ± 0.09d	4.63 ± 0.31c
18	HFr:HCh	8.05 ± 0.04d	4.70 ± 0.04c
19	HFr:Mam	8.07 ± 0.03d	4.72 ± 0.05c
20	MCh:HCh	9.43 ± 0.13a	6.18 ± 0.17a
21	MCh:Mam	8.51 ± 0.05cd	5.17 ± 0.06bc
22	HCh:Mam	8.89 ± 0.09bc	5.54 ± 0.11bc

Different letters in a column indicate significant differences at *p* < 0.05; statistically, a, b, c, and d following the values indicate significant differences among these values. Total polyphenols are expressed as TP. Total tannins are expressed as TTA.

**Table 4:** Color parameters of wines of *Vitis amurensis* (n = 3).

Sample	L*	a*	b*	c*	h*	ΔE*
Control	25.63 ± 0.07h	6.11 ± 0.35a	2.27 ± 0.03b	2.31 ± 0.04h	45.81 ± 0.23a	31.91 ± 0.36ab
NFr	28.15 ± 0.02a	4.64 ± 0.01b	1.83 ± 0.01b	4.98 ± 0.01cd	41.84 ± 0.01b	30.33 ± 0.02ab
MFr	26.32 ± 0.11fg	1.60 ± 0.21ef	2.24 ± 0.00b	2.76 ± 0.12gh	40.86 ± 0.42d	32.97 ± 0.24ab
HFr	26.5 ± 0.03ef	3.20 ± 0.03cd	2.18 ± 0.03b	3.89 ± 0.00ef	39.66 ± 0.07ef	32.18 ± 0.05ab
MCh	26.41 ± 0.08f	2.06 ± 0.14ef	2.20 ± 0.02b	2.81 ± 0.20g	39.91 ± 0.28e	32.71 ± 0.16ab
HCh	24.74 ± 0.02i	3.80 ± 0.06c	2.54 ± 0.02ab	2.57 ± 0.04gh	45.98 ± 0.18a	33.38 ± 0.07ab
MAm	26.84 ± 0.02de	4.63 ± 0.02b	2.47 ± 0.01ab	5.24 ± 0.01c	40.75 ± 0.01d	31.26 ± 0.03ab
NFr:MFr	25.43 ± 0.17h	3.25 ± 0.56cd	2.63 ± 0.01ab	4.19 ± 0.45ef	39.36 ± 0.06ef	32.95 ± 0.59ab
NFr:HFr	26.6 ± 0.01ef	2.64 ± 0.06de	2.09 ± 0.06b	3.37 ± 0.07f	39.06 ± 0.07f	32.34 ± 0.09ab
NFr:MCh	27.45 ± 0.04c	4.25 ± 0.15bc	2.10 ± 0.01b	4.74 ± 0.13d	40.96 ± 0.15cd	30.99 ± 0.16ab
NFr:HCh	26.76 ± 0.02e	5.42 ± 0.03a	2.34 ± 0.01b	5.90 ± 0.03b	41.76 ± 0.01c	31.1 ± 0.04ab
NFr:MAm	25.57 ± 0.09h	1.38 ± 0.08f	2.27 ± 0.08b	2.66 ± 0.03gh	41.37 ± 0.32cd	33.69 ± 0.14a
MFr:HFr	25.90 ± 0.18gh	3.52 ± 0.04cd	2.39 ± 0.05ab	4.26 ± 0.06e	39.59 ± 0.05ef	32.51 ± 0.19ab
MFr:MCh	28.01 ± 0.12ab	5.42 ± 0.27a	6.97 ± 6.54a	5.90 ± 0.23b	41.80 ± 0.32bc	28.74 ± 6.55b
MFr:HCh	27.86 ± 0.02b	6.00 ± 0.04a	2.12 ± 0.33b	6.37 ± 0.07a	42.63 ± 0.55b	30.02 ± 0.33ab
MFr:MAm	26.83 ± 0.06de	3.00 ± 0.16d	2.13 ± 0.08b	3.69 ± 0.08f	39.37 ± 0.33ef	31.99 ± 0.19ab
HFr:MCh	26.54 ± 0.11ef	4.11 ± 0.13bc	2.32 ± 0.00b	4.72 ± 0.11de	40.41 ± 0.14de	31.76 ± 0.17ab
HFr:HCh	26.11 ± 0.13g	2.88 ± 0.47de	2.22 ± 0.28b	3.65 ± 0.21f	39.68 ± 0.23ef	32.63 ± 0.56ab
HFr:MAm	27.06 ± 0.03d	2.87 ± 0.22de	1.91 ± 0.14b	3.45 ± 0.11f	39.57 ± 0.59ef	31.92 ± 0.26ab
MCh:HCh	25.68 ± 0.08h	2.42 ± 0.04de	2.39 ± 0.03ab	3.41 ± 0.01f	39.70 ± 0.07ef	33.13 ± 0.09ab
MCh:MAm	28.00 ± 0.11ab	2.23 ± 0.21e	1.83 ± 0.16b	2.89 ± 0.06g	39.31 ± 0.10ef	31.42 ± 0.29 ab
HCh:MAm	26.64 ± 0.04ef	2.95 ± 0.11d	2.36 ± 0.02b	3.78 ± 0.09f	38.94 ± 0.01f	32.10 ± 0.12 ab

Different letters in a column indicate significant differences at *p* < 0.05; statistically, a, b, c, and d following the values indicate significant differences among these values.

**Table 5:** Quantitative analysis of the aroma compounds of wines aged with oak chips. All values are expressed as means (μg/mL) ± standard deviation (SD).

Compounds	CON	NFr	MFr	HFr	MCh	HCh	MAm	NFr: MFr	NFr: HFr	NFr: MCh	NFr: HCh	NFr: MAm	MFr: HFr	MFr: MCh	MFr: HCh	MFr: MAm	HFr: MCh	HFr: HCh	HFr: MAm	MCh: HCh	MCh: MAm	HCh: MAm
Esters																						
Ethyl acetate	1.638 ± 0.15c	-	3.798 ± 0.54ab	1.723 ± 0.92c	-	-	-	-	-	5.345 ± 0.86a	-	-	-	-	-	2.738 ± 0.28cd	-	-	-	-	2.927 ± 0.54bc	-
Isopropyl acetate	37.60 ± 4.27gh	48.74 ± 3.30fg	52.54 ± 3.05f	37.80 ± 2.89gh	53.83 ± 1.65ef	59.94 ± 2.45ef	31.17 ± 3.33h	55.06 ± 0.95ef	71.89 ± 2.94d	83.8 ± 1.05c	43.69 ± 1.53g	47.13 ± 0.94fg	125.1 ± 1.45a	49.74 ± 2.72fg	48.18 ± 3.47fg	62.35 ± 3.53e	95.9± 3.10b	54.35 ± 0.93ef	46.5± 2.35fg	44.24 ± 3.66fg	57.45 ± 2.15ef	43.69 ± 2.46g
3-Methylbuty lacetate	5.635 ± 0.4ef	6.354 ± 0.81ef	8.08± 0.45de	5.513 ± 0.84ef	7.154 ± 0.42ef	8.14± 0.3de	4.847 ± 0.36f	7.978 ± 0.71de	9.26± 0.21d	14.72 ± 0.15c	5.353 ± 0.52f	5.943 ± 0.99ef	18.37 ± 0.54a	7.269 ± 0.39e	6.679 ± 0.42ef	8.87± 0.54de	15.35± 0.56bc	8.29± 0.45de	6.946 ± 0.47ef	6.213 ± 0.17ef	7.504 ± 0.96de	5.353 ± 0.3f
Butanoic acid, ethyl ester	2.271 ± 0.83b	2.669 ± 0.61b	2.648 ± 0.47b	2.694 ± 0.87b	2.891 ± 0.14b	2.855 ± 0.29b	2.042 ± 0.39b	3.098 ± 0.49b	3.736 ± 0.77ab	4.744 ± 0.39ab	2.458 ± 0.84b	2.508 ± 0.23b	5.725 ± 0.98a	3.201 ± 0.52b	2.644 ± 0.77b	3.811 ± 0.22ab	4.806 ± 0.24ab	3.048 ± 0.77b	2.592 ± 0.31b	2.991 ± 0.95b	2.618 ± 0.93b	2.458 ± 0.18b
Acetic acid, hydroxy-,ethyl ester	2.461 ± 0.68cd	9.77± 0.35a	3.680 ± 0.43c	4.680 ± 0.43bc	8.48± 0.82a	5.308 ± 0.75bc	1.240 ± 0.76d	3.998 ± 0.98c	4.860 ± 0.23bc	5.65± 0.21bc	2.967 ± 0.24cd	4.384 ± 0.38bc	6.030 ± 0.44b	4.382 ± 0.61bc	3.261 ± 0.29c	4.370 ± 0.74bc	4.481 ± 0.32bc	4.513 ± 0.13bc	3.814 ± 0.50c	3.130± 0.88c	4.243 ± 0.82bc	2.960 ± 0.17cd
Propanoic acid,2-hydroxy-, methyl ester	5.608 ± 0.28d	8.34± 0.57bc	7.092 ± 0.63cd	5.674 ± 0.59d	6.709 ± 0.52cd	7.733 ± 0.18c	5.691 ± 0.32d	7.86± 0.57c	9.50± 0.17b	10.85 ± 0.25ab	6.122 ± 0.56d	6.297 ± 0.22cd	12.22 ± 0.71a	8.49± 0.79bc	7.643 ± 0.54cd	8.44± 0.21bc	11.39 ± 0.57ab	8.19 ± 0.37bc	6.632 ± 0.38cd	7.264 ± 0.26cd	7.091 ± 0.69cd	6.122 ± 0.55d
Propanoic acid,2-hydroxy-, ethyl ester	2.264 ± 0.33bc	-	3.134 ± 0.12bc	-	-	2.872 ± 0.15bc	2.132 ± 0.79c	3.199 ± 0.18bc	4.011 ± 0.72b	-	-	-	-	6.993 ± 0.40a	-	3.683 ± 0.62bc	5.707 ± 0.65ab	3.411 ± 0.74bc	2.944 ± 0.94bc	-	-	-
Ethyl(s)- (-)-lactate	53.14 ± 1.92c	71.83 ± 2.36ab	56.36 ± 4.26bc	54.96 ± 1.57bc	56.86 ± 3.25bc	63.53 ± 3.77c	58.07 ± 1.86bc	72.59 ± 3.94ab	72.94 ± 4.35ab	71.96 ± 4.37ab	59.92 ± 2.86bc	59.55 ± 3.56bc	75.75 ± 1.24a	74.06 ± 1.69ab	67.84 ± 3.64ab	69.42 ± 1.39ab	72.01 ± 3.57ab	70.65 ± 3.25ab	67.42 ± 3.09ab	75.79 ± 1.13a	64.84 ± 3.81c	59.92 ± 2.13bc
Nonanoic acid, ethyl ester	3.545 ± 0.71d	5.006 ± 0.26d	5.051 ± 0.66d	4.353 ± 0.28d	4.303 ± 0.57d	5.876 ± 0.15cd	3.641 ± 0.33d	4.883 ± 0.77d	7.003 ± 0.47c	11.22 ± 0.57ab	3.938 ± 0.65d	4.183 ± 0.29d	12.47 ± 0.46a	5.811 ± 0.68cd	5.138 ± 0.42d	6.441 ± 0.14cd	9.98± 0.72b	5.197 ± 0.84d	4.317 ± 0.84d	3.731 ± 0.49d	4.548 ± 0.13d	3.938 ± 0.49d
Trans-2-hexenyl-acetate	11.69 ± 3.14c	16.51 ± 0.64bc	16.39 ± 0.97bc	13.52 ± 1.13c	14.30 ± 3.25c	18.69 ± 1.05bc	12.83 ± 3.46c	16.11 ± 0.89c	23.25 ± 2.16b	37.93 ± 0.97a	12.20 ± 2.31c	12.95 ± 2.18c	40.79 ± 2.03a	17.41 ± 1.87bc	15.89 ± 1.07c	20.74 ± 0.91bc	35.05 ± 3.30a	17.35 ± 2.49bc	14.15 ± 3.08c	11.79 ± 3.39c	15.41 ± 1.50c	12.21 ± 0.88c
Propanoic acid,3-methoxyl-, ethyl ester	34.11 ± 2.72b	9.30 ± 2.28de	37.27 ± 1.12b	14.43 ± 1.12de	14.89 ± 1.62d	9.19 ± 1.42de	5.203 ± 0.79e	9.42 ± 1.03de	54.07 ± 2.42a	11.52 ± 1.46de	24.71 ± 2.02c	8.33 ± 1.98e	13.79 ± 2.17de	10.07 ± 2.55de	7.531 ± 1.49e	9.64 ± 1.39de	12.82 ± 3.03de	13.06 ± 1.94de	8.19 ± 1.79e	7.660 ± 2.11e	34.54 ± 0.94b	24.71 ± 1.31c
Pentadecanoic acid, 3-methybutyl ester	80.3 ± 1.24f	131.1 ± 2.93cd	119.1 ± 4.28d	105.8 ± 3.03e	121.8 ± 2.02d	118.5 ± 3.15d	146.9 ± 1.28b	140.1 ± 2.29bc	157.2 ± 2.44a	137.9 ± 3.26bc	113.8 ± 1.09de	107.2 ± 2.49e	148.2 ± 2.17ab	140.3 ± 4.24bc	127.4 ± 3.98cd	138.3 ± 4.43bc	142.8 ± 1.19bc	142.7 ± 4.12bc	127.1 ± 1.09cd	143.2 ± 3.68b	133.6 ± 1.49c	113.8 ± 2.87de
Butanoic acid, diethyl ester	1.406 ± 0.22a	1.632 ± 0.30a	1.458 ± 0.44a	1.451 ± 0.33a	-	1.427 ± 0.57a	1.311 ± 0.22a	1.735 ± 0.41a	1.875 ± 0.32a	-	-	-	1.853 ± 0.64a	1.763 ± 0.94a	-	1.550 ± 0.41a	1.903 ± 0.41a	1.832 ± 0.77a	1.521 ± 0.63a	-	-	-
Benzoic acid, ethyl ester	2.729 ± 0.16b	2.647 ± 0.29bc	3.391 ± 0.42a	1.859 ± 0.21bc	2.749 ± 0.43a	2.963 ± 0.39a	1.293 ± 0.35c	3.148 ± 0.48a	4.814 ± 0.88a	4.046 ± 0.58a	1.812 ± 0.72c	1.997 ± 0.34bc	4.989 ± 0.87a	2.906 ± 0.92a	3.031 ± 0.85a	2.921 ± 0.79a	3.699 ± 0.37a	2.393 ± 0.89bc	1.052 ± 0.22c	2.068 ± 0.48bc	2.294 ± 0.63bc	1.812 ± 0.98c
Propanoic acid, 2-hydroxy-, ethyl ester	9.47 ± 1.87e	10.20 ± 1.99e	53.03 ± 3.39b	17.07 ± 1.19de	18.01 ± 2.26de	11.77 ± 1.14e	6.365 ± 1.46e	11.12 ± 2.13e	75.16 ± 3.33a	22.79 ± 1.12de	29.96 ± 1.24c	9.88 ± 2.55e	24.97 ± 2.88cd	10.55 ± 2.37e	8.21 ± 2.16e	11.63 ± 0.58e	22.06 ± 1.41d	12.50 ± 1.67e	9.12 ± 1.75e	6.691 ± 2.83e	47.82 ± 1.42b	29.96 ± 2.97c
Butanedioic acid, diethyl ester	47.76 ± 1.36b	51.31 ± 2.38b	0.921 ± 1.53d	44.47 ± 3.51bc	46.13 ± 1.48bc	0.980 ± 2.25d	39.75 ± 4.17c	57.98 ± 2.78ab	1.491 ± 1.09d	60.31 ± 2.24ab	46.14 ± 1.04bc	42.12 ± 3.58c	60.88 ± 3.87a	57.46 ± 2.86ab	49.81 ± 2.08b	56.42 ± 1.32ab	2.154 ± 0.34d	60.69 ± 4.19a	0.6981 ± 0.95d	58.03 ± 1.94ab	53.87 ± 2.97ab	46.14 ± 1.97b
2-Phenethyl acetate	14.41 ± 0.66e	16.20 ± 0.94de	16.82 ± 0.90d	11.45 ± 0.36f	13.36 ± 0.17ef	-	10.50 ± 0.72f	16.85 ± 0.65d	19.77 ± 0.54c	26.09 ± 0.53b	14.85 ± 0.47e	-	40.47 ± 0.33a	-	13.53 ± 0.13ef	-	-	15.84 ± 0.57de	-	12.28 ± 0.51f	15.54 ± 0.32de	14.85 ± 0.34e
Methyl dihydrojasmonate	6.025 ± 0.83de	6.585 ± 0.20d	5.150 ± 0.27ef	6.817 ± 0.99d	3.679 ± 0.23f	4.973 ± 0.73f	5.725 ± 0.84de	4.867 ± 0.74f	5.567 ± 0.67de	7.129 ± 0.11c	3.693 ± 0.19f	3.885 ± 0.94f	7.738 ± 0.76a	5.151 ± 0.78ef	4.593 ± 0.49f	5.646 ± 0.16de	7.179 ± 0.94b	6.132 ± 0.89de	5.435 ± 0.32e	4.582 ± 0.77f	5.144 ± 0.48f	3.693 ± 0.62f
Butanoic acid, hydroxy-, diethyl ester	90.4 ± 4.45e	1.591 ± 3.92g	1.741 ± 2.09g	90.1 ± 4.11e	92.7 ± 2.46e	92.4 ± 0.47e	126.5 ± 1.79ab	110.1 ± 2.50c	2.413 ± 0.89g	124.1 ± 1.43ab	-	81.9 ± 0.96f	128.2 ± 2.63a	111.2 ± 3.84c	101.1 ± 4.14d	109.9 ± 1.54c	6.183 ± 1.15g	120.9 ± 3.77b	1.701 ± 1.04g	110.5 ± 1.66c	108.1 ± 1.74cd	124.0 ± 0.75ab
Ethyl 3-methylbutyl succinate	38.05 ± 1.04cd	50.13 ± 1.54bc	43.06 ± 1.06c	37.99 ± 2.99cd	43.51 ± 2.16c	42.77 ± 3.22c	32.99 ± 1.00d	52.62 ± 1.16ab	59.45 ± 1.82a	54.76 ± 2.09ab	43.56 ± 2.10c	38.53 ± 1.23cd	58.63 ± 1.13a	55.05 ± 2.07ab	46.85 ± 1.82bc	50.68 ± 2.28bc	56.06 ± 2.27ab	55.49 ± 3.02ab	48.18 ± 0.94bc	54.33 ± 3.05ab	51.01 ± 3.44b	43.56 ± 3.45c
Tetradecanoic acid, ethyl ester	27.76 ± 1.19ab	30.25 ± 2.04ab	23.55 ± 1.82b	26.04 ± 1.42b	25.89 ± 2.28b	22.84 ± 3.08b	19.56 ± 3.01b	27.15 ± 1.73b	30.99 ± 1.96ab	26.58 ± 4.23b	22.63 ± 2.45b	19.72 ± 1.65b	29.71 ± 1.54ab	26.96 ± 2.77b	24.73 ± 0.91b	27.03 ± 3.82b	30.23 ± 2.40ab	35.14 ± 1.80a	26.39 ± 1.23b	26.17 ± 1.67b	29.47 ± 3.02ab	22.61 ± 1.92b
Hexadecanoic acid, methyl ester	2.023 ± 0.62a	1.732 ± 0.69a	1.345 ± 0.89a	1.894 ± 0.10a	1.361 ± 0.41a	1.382 ± 0.12a	1.343 ± 0.36a	1.813 ± 0.61a	1.708 ± 0.45a	2.156 ± 0.18a	1.561 ± 0.41a	1.064 ± 0.59a	2.407 ± 0.88a	1.633 ± 0.38a	1.531 ± 0.86a	2.254 ± 0.42a	2.677 ± 0.98a	2.069 ± 0.69a	1.564 ± 0.18a	2.928 ± 0.51a	1.792 ± 0.41a	1.561 ± 0.59a
Ethyl hydrogen succinate	116.0 ± 1.23j	161.8 ± 2.78h	157.1 ± 2.59hi	112.1 ± 1.63j	172.8 ± 3.96g	151.7 ± 1.77i	109.4 ± 4.03j	218.3 ± 2.51de	280.2 ± 1.98a	222.3 ± 1.06d	218.6 ± 3.89d	154.7 ± 2.46hi	206.7 ± 1.43e	250.6 ± 1.25bc	193.5 ± 4.35f	209.2 ± 3.85e	223.9 ± 3.23d	246.6 ± 3.13c	169.8 ± 2.33gh	248.8 ± 2.62c	246.2 ± 4.17c	218.6 ± 1.76d
Hexadecanoic acid, 2-hydroxyethyl ester	18.38 ± 1.01ab	22.98 ± 2.68ab	8.67 ± 3.54bc	21.84 ± 4.22ab	11.53 ± 2.05b	10.53 ± 4.46bc	23.68 ± 2.64ab	0.6435 ± 3.47c	13.80 ± 1.04b	27.39 ± 2.64a	18.79 ± 4.09ab	12.33 ± 3.42b	13.78 ± 2.38b	0.7617 ± 1.64c	19.99 ± 2.57ab	0.6411 ± 0.23c	18.93 ± 2.19ab	1.273 ± 1.14c	12.46 ± 2.08b	19.61 ± 4.26ab	16.68 ± 1.37b	18.79 ± 3.93ab
Total	614.7	666.7	631.4	624.2	722.9	646.4	652.2	831	915	973	676.8	624.6	1039	852	759.1	817	785.3	892	568.5	852	913	801
Alcohols																						
1-Propanol	97.9 ± 2.84h	141.4 ± 1.41g	125.2 ± 2.19fg	104.4 ± 2.17h	125.3 ± 3.21f	131.1 ± 1.09ef	82.7 ± 4.02i	154.1 ± 1.70cd	169.1 ± 3.21b	153.9 ± 3.43cd	121.3 ± 1.69fg	114.3 ± 3.32h	182.6 ± 4.21a	150.5 ± 1.84d	130.7 ± 2.89ef	158.9 ± 3.61c	165.9 ± 3.29b	136.2 ± 2.58e	126.8 ± 3.14ef	163.2 ± 1.66b	142.7 ± 3.31de	121.4 ± 3.76fg
1-Butanol	72.43 ± 2.33d	30.83 ± 2.06gh	84.8 ± 1.68c	46.04 ± 3.69fg	185.1 ± 3.87a	38.65 ± 3.61gh	41.88 ± 2.62fg	52.91 ± 3.61ef	105.2 ± 4.46b	46.86 ± 0.97fg	48.88 ± 4.07f	30.14 ± 1.49h	39.65 ± 2.35g	49.04 ± 4.37f	40.72 ± 2.02fg	53.41 ± 2.19ef	48.42 ± 1.15fg	45.69 ± 1.46fg	44.81 ± 1.57fg	60.85 ± 0.92e	84.3 ± 0.99c	48.88 ± 3.29f
1-Butanol,3-methyl-	167.4 ± 7.64d	201.3 ± 5.04bc	192.3 ± 6.76c	170.4 ± 8.49d	177.9 ± 5.90cd	195.1 ± 6.57bc	167.9 ± 9.49d	225.0 ± 5.21ab	237.3 ± 6.90a	203.4 ± 6.23bc	196.9 ± 5.64bc	188.2 ± 8.02cd	215.2 ± 6.54b	219.5 ± 5.85ab	201.1 ± 6.37bc	212.0 ± 8.12bc	170 ± 3.87d	202.9 ± 5.19bc	173 ± 3.37cd	215.9 ± 6.31ab	206.7 ± 6.83bc	196.9 ± 8.32bc
2-Pentanol	24.62 ± 2.57e	28.89 ± 4.08e	35.47 ± 3.27de	24.79 ± 2.08e	30.72 ± 3.93de	35.64 ± 2.42de	21.84 ± 3.28e	36.45 ± 1.65de	47.61 ± 4.32cd	67.74 ± 1.96b	23.54 ± 2.42e	26.61 ± 4.29e	81.3 ± 1.99a	32.78 ± 3.96de	30.19 ± 2.85de	39.76 ± 1.32d	55.61 ± 3.87c	37.16 ± 1.54de	31.81 ± 1.71de	25.37 ± 3.69e	33.86 ± 2.32de	23.54 ± 3.51e
1-Pentanol	57.68 ± 1.73gh	68.49 ± 3.35fg	82.3 ± 1.35ef	57.71 ± 2.91gh	70.66 ± 2.89fg	83.3 ± 1.56e	50.76 ± 3.01h	83.6± 2.68de	110.5 ± 2.74c	150.5 ± 4.24b	56.99 ± 4.27gh	62.45 ± 1.31g	177.4 ± 1.27a	75.81 ± 2.93ef	70.77 ± 2.50fg	92.8 ± 2.92d	156.1 ± 1.06b	88.7 ± 2.34de	73.47 ± 3.37f	61.15 ± 3.93g	79.81 ± 3.74ef	56.99 ± 2.31gh
4-Methylpentanol	2.987 ± 0.20b	-	3.309 ± 0.39ab	-	-	3.261 ± 0.41ab	2.951 ± 0.88b	3.936 ± 0.41ab	4.599 ± 0.63ab	-	-	-	4.823 ± 0.78a	3.639 ± 0.37ab	-	3.623 ± 0.21ab	4.339 ± 0.64ab	4.002 ± 0.60ab	3.242 ± 0.35ab	-	-	-
1-Hexanol	63.23 ± 1.39e	83.6 ± 1.65cd	70.61 ± 2.85de	67.18 ± 3.98e	76.43 ± 0.94d	79.73 ± 0.93cd	64.37 ± 2.67e	98.1 ± 2.65b	109.2 ± 1.94a	99.7 ± 1.33b	76.95 ± 2.55d	71.91 ± 1.38de	107.0 ± 1.88ab	94.2 ± 4.15bc	85.8 ± 4.29c	94.6 ± 2.47b	104.9 ± 2.06ab	96.9 ± 3.33b	86.0 ± 2.36c	98.5 ± 1.15b	81.3 ± 2.95cd	76.95 ± 2.57d
1-Hexen-3-ol	1.306 ± 0.82a	-	1.087 ± 0.56a	1.261 ± 0.43a	-	1.648 ± 0.85a	1.336 ± 0.47a	1.968 ± 0.88a	1.585 ± 0.16a	-	-	-	1.964 ± 0.59a	1.514 ± 0.73a	-	1.373 ± 0.38a	1.539 ± 0.17a	1.883 ± 0.59a	1.643 ± 0.59a	-	-	-
1-Propanol,3-ethoxy-	12.71 ± 0.81bc	11.79 ± 0.39cd	11.43 ± 0.88cd	9.74 ± 0.23d	10.34 ± 0.85cd	11.89 ± 0.87cd	9.16 ± 0.57d	13.72 ± 0.86bc	16.25 ± 0.74ab	16.59 ± 0.59a	11.56 ± 0.22cd	10.06 ± 0.48d	17.76 ± 0.60a	13.23 ± 0.28bc	12.15 ± 0.74c	13.72 ± 0.73bc	17.13 ± 0.68a	14.26 ± 0.25b	12.01 ± 0.39cd	13.52 ± 0.64bc	12.93 ± 0.31bc	11.56 ± 0.56cd
2-Hexen-1-ol	2.017 ± 0.12bc	-	2.737 ± 0.13bc	1.764 ± 0.92c	-	2.543 ± 0.67bc	1.539 ± 0.57c	2.493 ± 0.45bc	3.649 ± 0.19b	-	1.873 ± 0.51c	-	5.684 ± 0.65a	2.448 ± 0.69bc	-	2.717 ± 0.58bc	5.424 ± 0.82a	2.524 ± 0.16bc	2.168 ± 0.56bc	1.856 ± 0.29c	-	1.873 ± 0.26c
3-Hexen-1-ol, (*E*)-	3.771 ± 0.46cd	4.635 ± 0.73bc	5.086 ± 0.91bc	4.095 ± 0.81c	4.227 ± 0.95c	5.325 ± 0.94bc	3.468 ± 0.68cd	5.505 ± 0.23bc	6.981 ± 0.15b	10.56 ± 0.92a	3.474 ± 0.93cd	1.230 ± 0.80d	11.58 ± 0.49a	1.459 ± 0.48d	4.681 ± 0.46bc	5.793 ± 0.77bc	10.68 ± 0.86a	5.829 ± 0.39bc	4.834 ± 0.62bc	3.921 ± 0.54c	4.856 ± 0.98bc	3.474 ± 0.96cd
1-Octen-3-ol	17.82 ± 3.27cd	21.59 ± 2.52cd	23.58 ± 1.10c	18.04 ± 3.01cd	17.57 ± 0.28cd	25.31 ± 0.88bc	15.68 ± 2.56d	23.25 ± 3.14c	31.79 ± 2.74b	47.65 ± 2.13a	19.13 ± 2.36cd	19.21 ± 1.56cd	49.46 ± 2.81a	23.67 ± 3.23c	21.93 ± 1.29cd	27.53 ± 1.22bc	45.83 ± 2.34a	25.30 ± 0.71bc	20.86 ± 2.69cd	18.63 ± 2.39cd	23.36 ± 1.59c	19.13 ± 1.31cd
3-Hexen-1-ol, (*Z*)-	52.22 ± 3.03a	72.60 ± 2.10b	52.81 ± 1.97b	56.01 ± 1.74c	49.91 ± 1.08cd	74.79 ± 3.66cd	361.3 ± 3.89cd	84.2 ± 2.12cd	86.1 ± 3.62d	64.29 ± 4.13d	99.7 ± 1.72de	67.43 ± 3.43de	77.88 ± 1.16de	75.75 ± 2.06de	65.11 ± 3.59de	79.01 ± 2.49e	80.7 ± 1.83eg	61.28 ± 1.25eg	71.15 ± 0.97eg	69.75 ± 3.03eg	72.11 ± 3.97eg	99.7 ± 2.25g
1-Heptanol	5.210 ± 0.97a	-	1.398 ± 0.61b	-	-	0.7570 ± 0.88 bc	-	7.385 ± 0.54bc	14.05 ± 0.49bc	-	0.7511 ± 0.65 c	4.731 ± 0.17c	-	3.373 ± 0.32d	9.62 ± 0.85d	9.55 ± 0.58de	10.28 ± 0.96e	7.684 ± 0.73e	0.833 ± 0.29e	0.4410 ± 0.47 e	8.25 ± 0.91e	0.7514 ± 0.19 e
2,3- Butanediol, [R-(R*, R*)]-	33.63 ± 4.40a	43.82 ± 2.32a	40.21 ± 3.91ab	28.65 ± 3.71ab	39.52 ± 2.20ab	44.36 ± 1.62b	23.60 ± 2.21b	54.90 ± 2.69b	59.29 ± 3.23b	41.59 ± 2.01b	47.90 ± 1.26b	32.37 ± 0.97b	40.44 ± 2.80bc	51.34 ± 3.20bc	43.63 ± 4.14bc	54.02 ± 1.35bc	45.45 ± 3.31bc	60.23 ± 3.13bc	47.49 ± 2.71c	42.88 ± 4.32cd	38.64 ± 4.17cd	47.91 ± 3.27d
2-Octen-1-ol, (*E*)	13.76 ± 3.12a	16.44 ± 2.98a	19.79 ± 3.26a	13.90 ± 3.35b	15.69 ± 0.51bc	19.71 ± 2.94bc	11.53 ± 2.11c	19.17 ± 1.12c	27.66 ± 0.94c	40.64 ± 2.25cd	13.99 ± 2.81cd	14.27 ± 1.03cd	42.05 ± 3.18cd	17.82 ± 0.31cd	16.58 ± 1.14cd	21.51 ± 3.11cd	41.48 ± 3.41cd	21.58 ± 0.77cd	16.83 ± 1.53cd	14.33 ± 1.21cd	19.65 ± 3.25cd	13.99 ± 2.41d
À-terpineol	25.86 ± 1.48a	26.14 ± 2.29ab	25.31 ± 1.93ab	19.82 ± 4.44ab	29.97 ± 2.47ab	24.27 ± 1.04ab	23.64 ± 1.62ab	30.19 ± 1.26ab	29.87 ± 2.42ab	28.28 ± 1.50ab	22.33 ± 2.24ab	15.86 ± 3.62ab	38.72 ± 1.28ab	34.55 ± 1.20b	31.52 ± 3.57b	24.91 ± 4.42b	20.58 ± 1.01b	21.03 ± 1.29b	12.19 ± 2.24bc	26.12 ± 0.96bc	20.17 ± 2.57c	22.33 ± 1.31c
3-(Methylthio) propanol	37.72 ± 1.45a	50.09 ± 2.41ab	52.88 ± 3.86ab	36.75 ± 1.62b	43.51 ± 1.66bc	48.16 ± 4.06bc	36.73 ± 3.60bc	60.26 ± 1.73bc	73.88 ± 1.21c	63.67 ± 1.31c	1.880 ± 2.54	44.77 ± 4.24cd	59.04 ± 1.87cd	53.81 ± 2.33cd	50.48 ± 3.69cd	59.39 ± 0.96d	67.66 ± 2.49d	67.09 ± 1.70d	54.48 ± 4.23d	61.82 ± 2.41d	54.94 ± 1.29e	1.884 ± 1.31e
Benzyl Alcohol	1.012 ± 0.82a	1.672 ± 0.62ab	1.228 ± 0.53ab	1.194 ± 0.88ab	1.151 ± 0.63ab	1.822 ± 0.37ab	1.405 ± 0.2ab	2.411 ± 0.41ab	1.649 ± 0.72b	2.415 ± 0.31b	1.921 ± 0.73b	1.732 ± 0.39b	2.331 ± 0.67b	-	2.060 ± 0.38b	2.235 ± 0.68b	4.172 ± 1.33b	2.573 ± 0.52b	0.3870 ± 0.59 b	2.334 ± 0.94b	1.519 ± 0.66b	1.923 ± 0.26b
Phenylethyl Alcohol	127.9 ± 1.40c	137.9 ± 3.39bc	133.5 ± 3.76c	131.3 ± 2.84c	133.6 ± 1.32c	134.6 ± 1.12bc	129.4 ± 1.35c	141.0 ± 4.35bc	1439 ± 3.55a	140.9 ± 4.37bc	133.7 ± 1.55c	131.1 ± 1.10c	141.5 ± 1.32bc	141.1 ± 0.96bc	136.9 ± 2.32bc	140.7 ± 4.14bc	135.9 ± 0.71bc	142.8 ± 2.67b	110.3 ± 4.39d	141.8 ± 2.55bc	138.1 ± 2.68bc	133.8 ± 1.81c
Benzeneethanol,4-hydroxy-	65.33 ± 2.60f	94.5 ± 1.28d	78.53 ± 2.54e	80.0 ± 2.26e	84.6 ± 1.52e	92.4 ± 3.52de	65.70 ± 0.95f	120.9 ± 4.13bc	116.5 ± 3.83bc	111.6 ± 3.68c	115.3 ± 2.75bc	82.8 ± 3.12e	104.6 ± 2.33cd	116.5 ± 2.78bc	96.7 ± 4.36d	119.8 ± 4.12bc	116.8 ± 1.61bc	141.1 ± 1.52a	103.6 ± 4.39cd	122.4 ± 2.54b	111.7 ± 2.84c	115.3 ± 2.05bc
Total	886.5	1035.7	1043.6	873	1096.2	1054.4	1116.9	1221.4	1396.7	1290.3	998.1	919.2	1401	1162	1050.6	1217.4	1308.9	1186.7	998.4	1144.8	1134.9	998.3
Acids																						
Acetic acid	345.0 ± 2.68b	234.8 ± 2.32e	256.9 ± 1.11cd	205.1 ± 1.69h	260.8 ± 2.05cd	252.7 ± 2.59d	159.7 ± 4.05j	213.3 ± 3.68gh	170.5 ± 2.15i	223.8 ± 1.12f	201.4 ± 4.11h	153.5 ± 2.87j	244.1 ± 2.96d	264.6 ± 2.97c	218.2 ± 4.47fg	204.8 ± 3.42h	218.1 ± 2.55fg	4243 ± 4.35a	234.8 ± 1.04e	214.5 ± 2.29g	214.9 ± 1.53fg	201.4 ± 2.72h
Butyric acid	7.647 ± 0.16de	9.61 ± 0.68d	10.25 ± 0.25cd	7.573 ± 0.95e	8.50 ± 0.46de	-	6.408 ± 0.26e	10.89 ± 0.50cd	14.66 ± 0.49b	21.40 ± 0.89a	7.861 ± 0.44de	-	21.91 ± 0.10a	-	9.27 ± 0.47de	-	-	11.43 ± 0.97c	-	8.26 ± 0.45de	11.23 ± 0.46cd	7.861 ± 0.22de
Pentanoic acid	46.83 ± 3.22d	62.12 ± 3.98bc	53.61 ± 2.83c	48.75 ± 1.31c	50.98 ± 2.13c	-	46.18 ± 3.37c	72.96 ± 4.39b	75.31 ± 3.46ab	77.64 ± 2.78ab	58.19 ± 4.14c	-	5.750 ± 1.37c	-	63.84 ± 4.20bc	-	-	77.81 ± 3.59a	-	73.43 ± 2.99b	66.16 ± 1.13bc	58.19 ± 2.91c
Hexanoic acid	15.99 ± 0.69a	-	-	9.36 ± 0.67d	-	-	-	14.88 ± 0.34b	-	-	-	-	-	-	-	12.55 ± 0.33d	14.27 ± 0.67b	13.31 ± 0.95c	2.030 ± 0.66d	-	-	-
n-Decanoic acid	7.605 ± 0.16c	7.952 ± 0.51b	5.402 ± 0.76cd	5.748 ± 0.84cd	6.456 ± 0.19cd	5.681 ± 0.71cd	4.151 ± 0.41d	4.837 ± 0.64cd	11.24 ± 0.31b	7.849 ± 0.66bc	2.982 ± 0.13cd	3.771 ± 0.76d	11.69 ± 0.17a	4.746 ± 0.35cd	5.641 ± 0.33cd	7.841 ± 0.33bc	5.178 ± 0.46cd	10.43 ± 0.88b	4.642 ± 0.77d	-	5.282 ± 0.48cd	2.982 ± 0.95cd
n-Hexadecanoic acid	11.77 ± 1.19b	12.53 ± 1.91bc	13.04 ± 1.86bc	12.43 ± 0.90bc	13.27 ± 3.26b	-	6.534 ± 0.75b	13.92 ± 3.46b	21.30 ± 0.74b	12.39 ± 1.14bc	22.26 ± 2.13ab	-	13.32 ± 2.16b	-	12.05 ± 2.41bc	-	-	19.08 ± 1.29b	-	11.83 ± 0.86c	15.27 ± 1.17b	22.26 ± 2.41a
Total	434.8	327.0	339.2	289.0	340.0	258.4	223.0	330.8	293.0	343.1	292.7	157.3	296.8	269.3	309.0	225.2	237.5	356.4	241.5	308.0	312.8	292.7
Aldehydes																						
Furfural	-	1.092 ± 0.35a	0.915 ± 0.18a	0.885 ± 0.22a	0.930 ± 0.18a	0.845 ± 0.14a	1.088 ± 0.19a	0.880 ± 0.78a	1.312 ± 0.76a	1.816 ± 0.21a	0.7282 ± 0.64a	0.7009 ± 0.63a	0.842 ± 0.17a	1.097 ± 0.91a	0.7858 ± 0.73a	1.016 ± 0.15a	1.829 ± 0.19a	1.108 ± 0.35a	0.872 ± 0.76a	0.7372 ± 0.44a	1.004 ± 0.54a	0.7282 ± 0.89a
2-Furancarboxaldehyde,5-methyl-	2.101 ± 0.47a	2.342 ± 0.86a	1.829 ± 0.12a	2.030 ± 0.90a	1.936 ± 0.65a	1.898 ± 0.33a	1.773 ± 0.93a	2.496 ± 0.37a	2.745 ± 0.68a	2.931 ± 0.84a	2.221 ± 0.14a	1.778 ± 0.23a	2.571 ± 0.28a	2.398 ± 0.22a	2.188 ± 0.81a	2.445 ± 0.63a	3.033 ± 0.56a	2.779 ± 0.97a	2.158 ± 0.41a	2.471 ± 0.57a	2.343 ± 1.00a	2.221 ± 0.35a
Total	2.101	3.376	2.744	2.836	2.751	2.743	2.681	3.376	4.057	4.747	2.949	2.479	3.599	3.495	2.974	3.461	3.033	3.887	2.096	3.208	3.347	2.949
Volatile phenols																						
Phenol,2-methoxy-	2.788 ± 0.28c	-	3.426 ± 0.25bc	2.548 ± 0.81c	2.848 ± 0.17c	3.118 ± 0.78bc	2.057 ± 0.29c	3.323 ± 0.20bc	4.326 ± 0.14b	-	-	-	-	2.825 ± 0.22c	-	3.454 ± 0.29bc	6.492 ± 0.17a	3.626 ± 0.51bc	2.773 ± 0.36c	-	-	-
Phenol, 4-ethyl-	2.792 ± 0.88c	4.586 ± 0.67bc	2.962 ± 0.54c	4.550 ± 0.53bc	2.351 ± 0.47c	5.648 ± 0.93b	4.415 ± 0.48bc	7.429 ± 0.40ab	4.991 ± 0.62bc	7.867 ± 0.47ab	4.376 ± 0.34bc	4.691 ± 0.88bc	6.946 ± 0.48ab	6.264 ± 0.19b	5.688 ± 0.49b	7.221 ± 0.98ab	8.63 ± 0.59a	7.588 ± 0.14ab	6.684 ± 0.83ab	6.289 ± 0.58b	3.448 ± 0.97c	4.376 ± 0.36bc
Phenol, 4-ethyl-2-methoxy-	8.36 ± 3.02c	20.17 ± 2.02bc	8.75 ± 0.63c	12.99 ± 3.40c	8.33 ± 2.83c	22.99 ± 2.61ab	21.93 ± 3.38bc	30.99 ± 0.97a	13.65 ± 1.99c	30.14 ± 1.68ab	16.58 ± 1.38bc	19.69 ± 2.28bc	22.81 ± 2.13b	25.65 ± 2.48ab	25.03 ± 2.72ab	30.42 ± 1.01ab	28.26 ± 1.68ab	30.44 ± 3.06ab	29.25 ± 2.68ab	30.21 ± 2.41ab	10.72 ± 3.43c	16.58 ± 2.87bc
4-Viny phenol	2.548 ± 0.86a	1.363 ± 0.93a	1.701 ± 0.84a	1.584 ± 0.89a	2.198 ± 0.19a	1.162 ± 0.28a	1.132 ± 0.18a	1.337 ± 0.37a	1.832 ± 0.44a	1.808 ± 0.62a	0.900 ± 0.47a	0.7819 ± 0.78a	2.186 ± 0.82a	1.246 ± 0.21a	1.150 ± 0.66a	1.650 ± 0.43a	2.183 ± 0.61a	1.520 ± 0.88a	0.998 ± 0.27a	0.895 ± 0.33a	1.467 ± 0.39a	0.899 ± 0.14a
Phenol, 2,6-dimenthoxy-	13.58 ± 0.59a	6.170 ± 0.14cd	5.574 ± 0.91cd	8.65 ± 0.34b	6.320 ± 0.76c	5.513 ± 0.34cd	5.460 ± 0.75cd	4.410 ± 0.14cd	6.238 ± 0.63c	5.321 ± 0.18cd	2.817 ± 0.75d	3.160 ± 0.51d	8.70 ± 0.83b	4.730 ± 0.89cd	4.145 ± 0.67d	5.240 ± 0.65cd	6.831 ± 0.97bc	5.199 ± 0.91cd	5.440 ± 0.27cd	3.830 ± 0.17d	3.843 ± 0.81d	2.813 ± 0.15d
DL-a-Phenyllactic acid	5.826 ± 0.13c	6.587 ± 0.56bc	5.144 ± 0.16c	6.666 ± 0.82bc	5.206 ± 0.87c	5.375 ± 0.14c	4.529 ± 0.42c	5.809 ± 0.19c	6.601 ± 0.51bc	7.034 ± 0.24b	5.046 ± 0.48c	4.639 ± 0.59c	10.21 ± 0.59a	6.044 ± 0.64c	5.095 ± 0.81c	6.310 ± 0.25c	8.32 ± 0.25ab	6.414 ± 0.83c	5.391 ± 0.65c	5.832 ± 0.41c	6.086 ± 0.99c	5.046 ± 0.75c
2,4-Di-tert-butylphenol	45.22 ± 3.50cd	47.52 ± 1.31cd	45.86 ± 4.12cd	38.57 ± 2.45de	42.33 ± 2.51cd	40.36 ± 2.85d	34.91 ± 1.45de	52.61 ± 3.42bc	59.99 ± 3.18b	67.84 ± 3.07ab	40.11 ± 3.03d	33.49 ± 4.45de	69.80 ± 2.62a	49.21 ± 1.84cd	51.16 ± 0.99bc	47.95 ± 1.07cd	50.82 ± 1.04c	50.86 ± 1.85c	30.34 ± 1.81e	46.01 ± 4.48cd	47.67 ± 1.95cd	40.11 ± 1.96d
2,3-Dimethylphenol	12.55 ± 1.36ab	10.32 ± 1.51ab	9.69 ± 0.66ab	14.38 ± 3.09a	11.40 ± 2.74ab	7.762 ± 1.49ab	-	7.320 ± 0.51ab	10.83 ± 0.54ab	10.02 ± 2.64ab	6.287 ± 3.01b	4.583 ± 2.36b	13.82 ± 2.88ab	8.09 ± 2.85ab	5.913 ± 2.32b	10.67 ± 2.75ab	10.84 ± 2.66ab	9.38 ± 2.65ab	7.141 ± 0.96b	5.700 ± 1.28b	13.20 ± 3.40ab	6.281 ± 0.58b
Total	93.7	96.7	83.1	89.9	81.0	91.9	74.43	113.2	108.5	130.0	76.12	71.03	134.5	104.1	98.2	112.9	122.4	115.0	88.0	98.8	86.4	76.11

Different letters in a column indicate significant differences at *p* < 0.05; statistically, a, b, c, and d following the values indicate significant differences among these values.

**Table 6:** Sensory evaluation of wines after oak chip additions.

Sample	Appearance	Aroma	Taste	Typicality	Clarity	Total Scores
	3	6	6	3	2	20
Control	2.1	4.4	4.3	1.5	1.4	13.7
NFr	2.4	4.8	4.6	1.4	1.6	14.8
MFr	2.3	4.8	4.5	1.7	1.5	14.8
HFr	2.4	4.5	4.4	1.5	1.6	14.4
MCh	2.5	4.5	4.5	1.8	1.7	15.0
HCh	2.5	4.2	4.3	1.9	1.5	14.4
MAm	2.3	4.6	4.4	1.5	1.6	14.4
NFr:MFr	2.4	4.7	4.8	1.4	1.6	14.9
NFr:HFr	2.5	4.8	4.4	2.0	1.4	15.1
NFr:MCh	2.6	4.8	4.4	1.8	1.5	15.1
NFr:HCh	2.3	4.7	4.2	1.7	1.6	14.5
NFr:MAm	2.3	4.6	4.7	1.8	1.5	14.9
MFr:HFr	2.2	4.9	4.5	1.9	1.5	15.0
MFr:MCh	2.5	4.4	4.3	1.7	1.8	14.7
MFr:HCh	2.3	4.6	4.5	1.6	1.5	14.5
MFr:MAm	2.6	4.5	4.5	1.9	1.6	15.1
HFr:MCh	2.2	4.8	4.4	1.8	1.4	14.6
HFr:HCh	2.6	5	4.2	1.4	1.4	14.6
HFr:MAm	2.4	4.8	4.1	1.8	1.5	14.6
MCh:HCh	2.5	4.7	4.3	1.7	1.5	14.7
MCh:MAm	2.2	4.7	4.8	1.7	1.6	15.0
HCh:MAm	2.4	4.6	4.3	1.6	1.7	14.6

## Data Availability

Not applicable.

## Supplementary Materials

The following supporting information can be downloaded at: https://www.mdpi.com/article/10.3390/foods11081126/s1, Table S1: Calibration curves for quantification in this study; Table S2: Quantitative standards and calibration curves for quantification of volatile compounds in this study; Table S3: Electronic tongue data of wine made from Vitis amurensis(n = 3).
